# Practicing Psychiatry in the Transitional Period: Lessons Learnt and Issues of the Steps Towards the New Normal

**DOI:** 10.1192/j.eurpsy.2022.50

**Published:** 2022-09-01

**Authors:** M. Al-Uzri

**Affiliations:** The Royal College of Psychiatrists, International Team, London, United Kingdom

## Abstract

The arrival of Covid-19 had huge impact on all aspects of life and psychiatry was no exception. Significant changes to mental health services were introduced to reduce the risk of infection among the inpatient population, patients in the community and in outpatients consultations. Use of virtual consultations in the community and stringent infection control in inpatient setting are examples of many. Those changes were associated with significant differences in patients’ presentation and access to services. Multiple studies reported increased proportion of psychotic patients being admitted to inpatient care. However, there were no reports on increased suicide contrary to the expectation. This is despite increased reports of mental distress, depression, and anxiety in the wider population. These reports are consistent with our knowledge of mental health problems reported in times of wars and disasters. Hence, some of the learning from experience proved relevant when facing this pandemic. Unfortunately, Covid-19 continues to represent a challenge and relaxing of the restrictions introduced were short-lived with every variant reported. In addition, some of the changes adopted by mental health services proved to be innovative and more efficient way of delivering care in psychiatry. Therefore, the early indications suggest that some of those changes would become the new normal.

**Disclosure:**

No significant relationships.

